# Performance of power generation: A dynamic productivity and efficiency analysis^☆^

**DOI:** 10.1016/j.heliyon.2024.e33112

**Published:** 2024-06-15

**Authors:** Reza FathollahZadeh Aghdam, Sami Al-Kharusi, Nisar Ahmad, Adham Al-Said, Bahareh Berenjforoush Azar

**Affiliations:** a Department of Economics & Finance Sultan Qaboos University, Oman; b University of Technology Sydney, Australia

**Keywords:** Electricity generation, Desalination firms, Malmquist index, DEA, Total factor productivity decomposition, Technological change, Efficiency change, COVID-19

## Abstract

This study evaluates the performance dynamics of Oman's principal part of electricity generation. Emphasis is placed on capturing the influence of the COVID-19 pandemic on the industry's performance indicators. We utilised the Malmquist Index method to analyse the changes in overall performance indicators over time. This approach allows us to distinguish between efficiency and technology changes. In addition, we employed the analysis of variance approach to test hypotheses related to COVID-19. Data is collected from twelve electricity producers across Oman's power sector. These consist of companies listed on the Stock Exchange Market in Oman, accounting for about 60 % of the total electricity production in the Sultanate. The predominant findings indicate that COVID-19 detrimentally impacted the sector's aggregate performance in 2020 but had a swift recovery in 2021. The analysis of the sample firm's productivity indices verifies that the decline in productivity in 2020 due to the Pandemic is attributable to a decrease in average efficiency indices and a negative shift in the projected frontier. These are probable consequences of the recessionary effects caused by the Pandemic. Despite a considerable decrease in average efficiency scores, a positive change in the frontier has facilitated a rapid recovery in 2021. This recovery can be attributed to the implementation of advanced technical upgrades in particular enterprises, which began as early as 2018/2019, well before the onset of the Pandemic.

## 1 Introduction

Understanding the dynamics of the performance of the Electricity Sector Industry (ESI) or its components is of immense importance for policy making and strategic development in every country, including Oman. A reliable and affordable electricity supply is an integral component of the Sultanate's strategic development. Oman's fast-growing electricity demand requires adequate infrastructure investments [1]. Consequently, ensuring the efficient performance of ESI is identified as one of the core objectives of the Authority for Public Services Regulation (APSR). Alongside other objectives, this objective is one of the prime goals of ASPR after the release of Vision 2040, recently launched by the Omani government, which sets out a clear and transparent strategy for national transformation and modernisation of the country (ASPR [2].

**Fig. 1:**
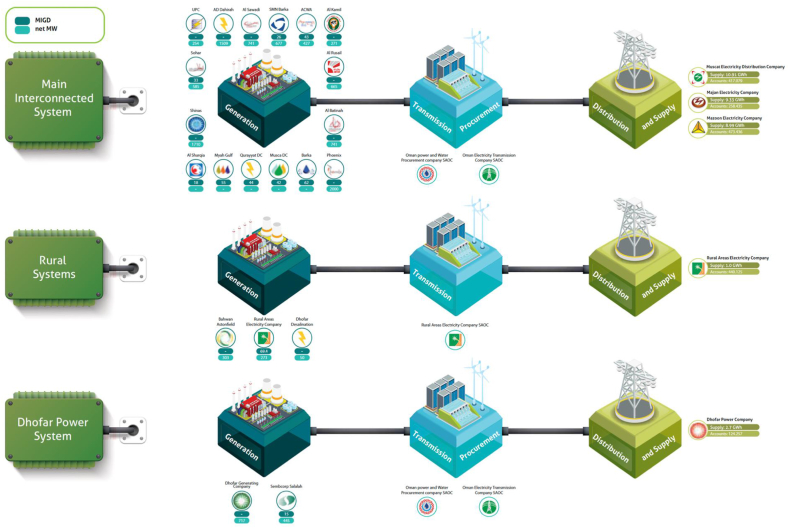
The Structure of Power System in Omani ESI (Source: APSR 2021). **Note:** Selected firms include: (i) Acwa Power; (ii) Al Kamel Power; (iii) Al Suwadi Power; (iv) Albatinah Power; (v) Sembcorp Salalah; (vi) Sharqiyah Desalination; (vii) SMN Power; (viii) Sohar Power; (ix) Phoenix Power; and (x) MCT Desalinate; (xi) MCT Desalinate; and (xii) Musandam Power. These firms collectively produced far above 60 % of electricity generated in Oman (APSR 2021) and have sample firms from all three regions of Oman's Power System (i.e., MIS, DPS, and RAEC).

The ESI in Oman has undergone significant market reform since 2004, making the sector compatible with worldwide standards. The modern ESI structure is now dominantly a market-oriented business which is functionally unbundled to generation, transmission, distribution, and market procurement segments. The generation segment is run by fully privatised firms or what are known as Independent Power Producers (IPPs)—predominantly listed in the Muscat Stock Exchange market. A competitive *single-buyer model* allows the generators to bid for selling their electricity production to a central procurement authority (the market operator). At the same time, bilateral sales are also permitted under the regulatory authority's supervision. Under the restructured ESI, independent regulatory authority was established in 2004 under Royal Decree 78/2004 to regulate the ESI and some aspects of the water sector. It was initially titled the Authority of Electricity Regulation (AER). Later in 2017, the government assigned some additional responsibilities, and it was eventually renamed the Authority for Public Services Regulation (APSR) under Royal Decree 78/2020. Fig. 1 best demonstrates the current structure of the Omani ESI.

Due to geographical conditions, the Sultanate of Oman's ESI and related water sector is divided into three distinct market segments, namely the Main Interconnected System (MIS) in the north, the Dhofar Power System (DPS) in the south, and the Rural Areas Electricity Company (RAEC) in segregated areas. As of 2021, the MIS accounts for 88 % of the total electricity supplied to Oman. The DPS holds 9 % of the electricity provided to the Sultanate, while the RAEC comprises only 3 % (ASPR 2021).

In the MIS region, at present, there are 16 power-generating companies. The generation segment in MIS is now entirely owned by the private sector (Hasan, et al. [3]. There is also one transmission company, one procurement company, and three supply and distribution companies. The transmission company is Oman Electricity Transmission Company (OETC), while the procurement company is Oman Power and Water Procurement Company (OPWPC). Three supply and distribution companies include Muscat Electricity Distribution Company (MEDC), Majan Electricity Company (MJEC), and Mazoon Electricity Company (MZEC). Significant efforts are undergoing to privatise transmission and distribution companies. If implemented, Oman will be the first country in the Middle East to privatise its transmission and distribution sub-sectors [3].

The DPS region has two prominent power-generating companies, the Dhofar Generating Company (DGC) and Sembcorp Salalah, both listed in the Muscat Stock Exchange Market. The transmission and procurement companies in the DPS are the ones in the northern region of MIS (i.e., OETC and OPWPC). In addition, there is one distribution and supply company. (i.e., Dhofar Power Company (DPC)). By the end of 2013, the ASPR (formerly AER) completed the relevant regulatory framework to ensure that the market structure in the DPS would be like the one in the MIS, effective from January 1, 2014. DPC has a license to distribute and supply in the new DPS market structure. The generation assets were transferred to Dhofar Generation Company (DGC) as an IPP, and transmission assets were transferred to OETC [1]. It is expected that DPS will be integrated with MIS by 2023 through a 400-kV transmission line, resulting in more operational efficiencies for the Omani ESI and allowing for more effective utilisation of the upcoming renewable energy capacities (wind and solar) of the southern parts of Oman [3].

The RAEC region includes very separate areas of Oman, including the Musandam peninsula, located south of the Strait of Hormuz. The land connection between Musandam and Oman's mainland is only through the United Arab Emirates (UAE). The leading independent power producers (IPPs) in the RAEC region are Musandam Power (listed in Muscat Exchange Market—not shown in Fig. 1) and Bahwan Astonfield Solar. RAEC SOAC owns this region's transmission and distribution entities (see Fig. 1).

Oman's ESI is also interconnected to the Gulf Cooperation Council (GCC) network. This is through limited transmission capacity with the UAE (Abu Dhabi), allowing implicit connection to the isolated Musandam area. Further, a direct transmission line between Oman and Saudi Arabia is likely to prevent the reliance on UAE's grid for future electricity exchanges with other GCC countries [3]. The total net electricity production was approximately 11,735,036 MWh, and the total net desalination water production was around 101,065,000 m^3^ during the second Quarter of 2021 [2].

It is vital to analyse the impacts of any structural changes in Oman's ESI on its performance. For instance, apart from market reform, it is crucial to capture the implications of the COVID-19 Pandemic on the industry's overall performance or its components, which ultimately contribute to Oman's macroeconomic performance. The critical questions are: How is power generating in Oman impacted by COVID-19? Moreover, what has been the magnitude of such an impact? How could researchers systematically and objectively capture the dynamics of firms' performances in Oman's ESI? What are the policy implications?

Against the above background, the prime objective of this paper is to examine the dynamics of productivity and efficiency changes in the generation segment of Omani ESI. Special attention is made to capturing COVID-19 impact on the performance of power generations in Oman. We presented the preliminary results of this study at the 44th IAEE International Conference [4], but this study has extensive results. To the best of our knowledge, this is the first study of its kind for Oman (See Section 2—the literature review).

The rest of the paper is structured like the following. Section 2 is a concise, systematic literature review on the productivity of ESIs. Section 3 justifies and describes the methodology implemented in this paper. Section 4 represents the data structure. Section 5 provides the results. Section 6 concludes and provides policy implications.

## 2 Literature review

This Section presents a brief systematic literature review (SLR) of earlier studies on productivity and efficiency analysis of ESI worldwide. The methodology used in this SLR is similar to Asatullaeva, et al. [5] and Ahmad, et al. [6], to name a few. This methodology has recently emerged in almost every area of research. It is objective and replicable, targeting the best relevant and most influential articles in revealing research gaps in a specific area of research. The relevant articles are searched in the Scopus database using a comprehensive list of words in the document's title, abstract and keywords.^1^ This initial search identified 362 documents. In the next step, we restricted our sample to the subject area of business, economics, econometrics, and finance according to Scopus subject classifications which removed 100 articles. To consider the quality of the paper, we then only considered journal articles. Finally, the articles written in languages other than English are removed. The sample at this stage consists of 221 articles. The remaining articles were manually screened for relevancy defined by the scope of the current paper. After filtration, the final sample consists of 203 journal articles published in 82 journals, including Sueyoshi and Goto [7], Walheer [8], Han, et al. [9] to name a few relevant articles.

Our literature review reveals that the most relevant journal on this topic is *Energy Economics*, with 34 publications, followed by *Energy Policy* and *Energy,* with 21 and 13 publications, respectively. Goto M., and Cullmann A., are the most productive authors, with six publications each. The most cited article is Sueyoshi and Goto [7] with 222 total citations. We did not find any study explicitly focusing on measuring productivity or efficiency for any GCC countries. However, we found only a one-panel study that included Bahrain and Saudi Arabia [10] in its country samples. This observation is the only among GCC countries out of 126 countries studied in the selection of 203 papers. Therefore, the current study will be the first study on Oman, opening doors for further research and collaborations in this area of research in the GCC region.

In Section 3, it is justified that this study adopts the *DEA-like Malmquist index* method. Thus, we provided the detailed contents of empirical studies using the Malmquist index in Table 1. We have identified only 18 studies out of 203 papers using the Malmquist Index for ESIs. These studies are the most relevant peers to our research. The detailed content analysis of these papers has been carried out to determine the type of efficiency, methods used, country of research, the study's time span, decision-making units (DMUs), and input and output variables. The articles reported in the Table are sorted based on each paper's average citations per year (ACPY). Nakano and Managi [11] is the most cited article in this context, with nearly seven citations per year since its publication.

**Table 1:** Empirical Studies of efficiency measurement using Malmquist index.

Rank	Reference	ACPY	Year	Type of efficiency	Methodology	list of countries	Period	# of DMU	DMU	Output variable	Input variable
10	Nakano and Managi [11]	6.9	2008	Technical	DEA, Malmquist, TFP	Japan	1978–2003	10	Firm	Electricity Production	Labor, Fuel, Capital
22	Yaisawarng and Klein [21]	5.1	1994	Technical, Scale	DEA, Malmquist	US	1985–1989	61	Plant	Electricity Production	Labor, Fuel, Capital, Sulphur
24	Monastyrenko [17]	4.7	2017	Eco-efficiency	DEA, Malmquist	Germany, France, UK, Italy, Portugal, Spain, Czech Republic, Poland, Benelux and Nordic EU countries	2005–2013	129	Firm	Electricity Production, CO2 Emission	Labor, Fuel, Capacity
26	Førsund and Kittelsen [22]	4.3	1998	Technical	DEA, Malmquist	Norway	1983–1989	150	Firm	Energy Production, Customers, Distance Index	Labor, Capital, Material, Energy loss
34	Walheer [8]	4.0	2019	Technical	DEA, Malmquist	US	2000–2012	2000	Plant	Electricity Generation	Fuel, Capacity
75	Aghdam [15]	2.2	2011	Technical	Malmquist, TFP, ANOVA	Australia	1969–2007	39	State	Electricity Generation, Electricity Consumption	Labor, Fuel, Capacity, Network Length, Network Capacity,
78	Miguéis, et al. [23]	2.1	2012	Technical	DEA, Malmquist, TFP	Norway	2004–2007	127	Firm	Energy Delivered, Customers	Labor, Capital, Energy loss, Material
79	Wang, et al. [24]	2.1	2007	Technical	DEA, Malmquist, TFP	Hongkong	1995–2003	2	Plant	Sales	Labor, Capital
81	Mirza, et al. [25]	2.0	2021	Technical	Malmquist, TFP, SFA (Translog Distance function)	Pakistan	2006–2016	8	Firm	Energy Delivered, Customers	Distribution losses, network length, peak load
98	Sánchez-Ortiz, et al. [26]	1.7	2020	Technical	DEA, Malmquist, TFP	Spain	2006–2015	5	Firm	Sales, Energy Sales, Interruption time	Labor, Capacity, Energy, Operating Expenses
110	Zhu, et al. [18]	1.3	2019	Productive	DEA, Malmquist	China	2005–2014	30	Province	Power production, CO2 emission	Labor, Fuel, Capacity
114	Cullmann and Von Hirschhausen [20]	1.2	2008	Technical	DEA, Malmquist, SFA (Translog Production function)	Poland	1997–2002	32	Firm	Sales, Customers	Labor, Capital, Network length
119	Li, et al. [13]	1.0	2022	Technical	DEA, Malmquist	China	2016–2019	30	Province	CO2 emission	Labor, Capital, Land, Energy
125	Singh, et al. [27]	1.0	2013	Technical	DEA, Malmquist, TFP	India	2003–2010	25	Plant	Electricity Production	Coal, Oil, Capacity, Auxiliary Power, Operational Time
138	Tai wu, et al. [14]	0.6	2010	Technical	DEA, Malmquist, TFP	China	1999–2007	30	Province	Electricity Generation	Labor, Fuel, Capital, SO2 Emission
140	Senyonga and Bergland [28]	0.6	2018	Technical	Malmquist, TFP, SFA (Translog Distance function)	Norway	2004–2012	121	Firm	Energy Delivered, Customers, Voltage lines	Capital, Operating Expenses
180	Paul and Shankar [16]	0	2022	Technical	Malmquist, TFP, SFA (Translog Production function)	Australia, Austria, Belgium, Canada, chez Republic, Denmark, Finland, France, Germany, Greece, Hungary, Ireland, Italy, Japan, Luxembourg, Mexico, Netherlands, Poland, Portugal, Slovak Republic, Spain, Sweden, Turkey, United Kingdom, United States	1980–2013	25	Country	Electricity Generation	Labor, Fuel, Capital, Capacity
201	Hon, et al. [29]	0	2014	Technical	DEA, Malmquist, TFP	Malaysia	1993–2008	3	Firm	Electricity Generation	Labor, Capacity

As shown in Table 1, nine studies have been carried out on firm levels as DMU. There are four studies with power plants as DMU. Three studies have been done at the provincial level for China [[12], [13], [14]]. There is only one study doing it at the state level for Australia Aghdam [15] and one country-level study for 25 OECD countries [16]. Most of the studies have been estimating total factor productivity (TFP) and technical efficiency (TE), except one for eco-efficiency [17] and one study focusing on productive efficiency [18]. The prime approaches used in estimating the Malmquist index have been a combination of DEA [11] SFA [19] or both [20].

As shown in Table 1, the time span of all the studies is pre-COVID. Therefore, the current research will also be the first to compare the firms' productivity and efficiency before and during the Pandemic. Electricity production (generation) is a widely used output variable. Other output variables include sales [24], customers and distance index [22,23], and CO2 emission [17], interruption time [26] etc. The most common input variables include labour, capital and fuel. The other input variables are material and energy loss [22], installation capacity [8,15,17], and operating expenses [28].

The most cited paper is Nakano and Managi [11] which has received 103 Scopus citations since its publication in 2008. The paper measures productivity in Japan's steam power generation sector. Further, it examines the effect of reforms on the productivity of 10 firms in this sector from 1978 to 2003. The study estimates a generalised form of the Malmquist productivity index using the DEA approach. In the next step, the study also assessed the impact of different factors associated with productivity change using the GMM panel data method. The study's main finding is that the regulatory reforms have contributed to productivity growth in Japan's steam power generation sector.

This study's main contribution will be using the *DEA-like Malmquist index* methodology like the above 18 studies in the context of Oman's power-generation sector. It further offers four distinct input-output configurations for this purpose, analysing critical aspects of TFP and efficiency changes in this sector. Further contributions will be related to the relevant policy implications of the analysis.

## 3 Methodology

Measuring the performance of firms/DMUs such as power plants, power generating firms, transmission and distribution firms, or the entire ESI have several well-established approaches. They are calculating some partial or total factor productivity (PFP/TFP) measures or efficiency indicators. One attractive method is benchmarking each firm's performance against its peers (similar domestic or international firms). This relative analysis is prevalent due to its objectivity—also known as the *frontier approach*, explicitly distinguishing between *productivity* and *efficiency* concepts. In contrast, these concepts are often used interchangeably in layperson language.

The *productivity* is often measured by an *index* number (usually unit free), reflecting the overall performance of a firm over time. For instance, higher productivity refers to a state where, given specific inputs, more output can be produced—alternatively, a case where, given specific output, lower inputs are used. In contrast, the *efficiency* score technically reflects how poor a firm performs relatively against the industry's best practice, known as the *frontier*. In other words, the *frontier*, at each period, divides the whole set of input-output data points between what can practically be attained and what cannot, given the current technology. Firms located on the frontier in a specific period are considered technically efficient, with an efficiency score of one. They are the peers for capturing efficiency scores of those away from the frontier (yet in the attainable area), often measured by a radial distance indicator—a distance function. They are hence identified as *inefficient* firms.

In frontier analysis, there are two principal schools of thought: Data Envelopment Analysis (DEA) versus Stochastic Frontier Approach (SFA). DEA is a non-parametric and, in most cases, deterministic methodology, making no account for the data noise. The DEA is non-parametric because there is no need to assume any parametric functional form for the shape of the frontier (e.g., production, cost, or profit functions). Instead, it uses Linear Programming (LPs) to determine a piece-wise envelopment frontier function. Such an enveloped frontier—a kinked linear function—distinguishes between what can or cannot be produced, given the existing technologies. The extent of distances (often radial distance from the origin) captures efficiency/inefficiency scores. Efficiency scores vary between zero (perfectly inefficient) and one (most efficient) at each period.

Alternatively, the Stochastic Frontier Approach (SFA) is parametric and stochastic (non-deterministic), allowing for data noise. The SFA is an advanced econometric methodology using two different residual terms in its pre-specified functional forms. Like every econometric Model, one residual term is assumed to be a normally distributed variable. At the same time, the other one holds a truncated distribution, taking a value between zero and 1, indicating whether a firm is on the frontier or not. One of the most stated shortcomings of the SFA is its reliance on the pre-specification of a functional form that may be subject to errors. There is a vast literature on *frontier analysis* using DEA or SFA approaches [30].

This study utilises the *DEA-like Malmquist Index* technique. It is justified because it allows the decomposition of variations in TFP indices into changes caused by relative efficiency changes (proximity of each firm to estimated best practice frontier over time). Further, it identifies how much of a TFP change is due to shifts that have taken place in the frontier as time passes. This latter component of TFP change represents technological changes (see Coelli, et al. [30]). Inward shifts usually resemble a recessionary downfall in the economy. In contrast, the outward shifts may occur during economic booms or whenever genuine technological innovations emerge (i.e., an outward shift that makes never attained data points attainable). The latter is more critical in every economy because it reflects a technological advancement that benefits everyone.

This technique (i.e., *DEA-like Malmquist Index*) is articulated in Coelli, et al. [30] (pp. 289–310), using the idea of Distance Function. The *DEA-like Malmquist Index* can be estimated as either output- or input-oriented. It is correct to choose each estimation method as long as we remain consistent in our selection alongside our study. The output-oriented Model is formulated as follows (see (Coelli, et al. [30], pp. 47–51).

(Equation 1) $$m_{o}\left(y_{t-1},x_{t-1};y_{t},x_{t}\right)=\frac{d_{o}^{t}\left(y_{t},x_{t}\right)}{d_{o}^{t-1}\left(y_{t-1},x_{t-1}\right)}\times {\left[\frac{d_{o}^{t-1}\left(y_{t},x_{t}\right)}{d_{o}^{t}\left(y_{t},x_{t}\right)}\times \frac{d_{o}^{t-1}\left(y_{t-1},x_{t-1}\right)}{d_{o}^{t}\left(y_{t-1},x_{t-1}\right)}\right]}^{1/2}$$

In this formula, *x* and *y* refer to the vector of inputs and outputs, respectively, and *m*_*o*_ shows the change in the TFP level. The formula indicates several distance functions, marked by the symbol *d*_*o*_*(.)*. For example, $d_{o}^{t-1}\left(y_{t},x_{t}\right)$ is the distance of a representative firm at year *t* from the frontier at year *t-*_*1*_. A value of *m*_*o*_ less than one shows a decline in TFP level from year *t-1* to year *t*.

**Fig. 2:**
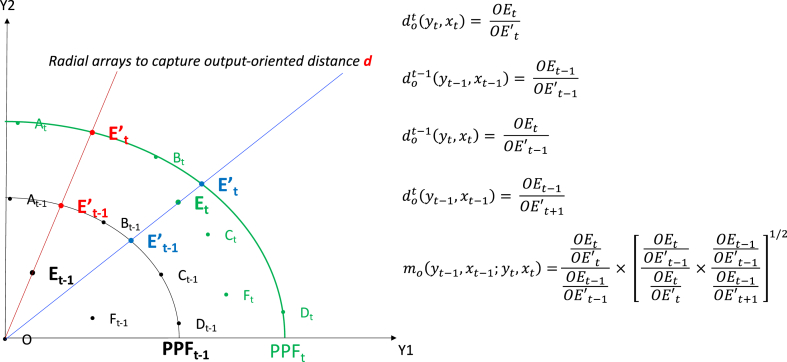
Output-Oriented Production Possibility Frontiers (PPF) in time *t* and *t-1*: Given inputs used.

Fig. 2 best visualises the (output-oriented) *DEA-like Malmquist Index* model of Equation (1) by representing two consecutive frontiers at *t-1* and *t* —**PPF**_**t-1**_ and **PPF**_**t**_. The data points of five firms (i.e., A, B, C, D, E and F) are shown in these two periods, specified by time-subscripts *t-1* or *t*. The firms on each period's frontier are those which, given the technology and their level of inputs, relatively produced the highest outputs in that period. These include firms A, B, C, and D in time *t-1* and firms A, B, and D in time *t*. Firms which are depicted below the PPFs are considered *inefficient* firms. For example, firms E and F are inefficient in time *t-1*, while firms C, E, and F are inefficient in time *t*.

In Fig. 2, two radial arrays are sketched from Firm E's data points in time *t-1* and *t*, respectively, to the origin (point O) to assign this firm's relative and consistent efficiency score in each period, also known as the *distance function*—*d*_*o*_*(.)*. For this purpose, hypothetical points E't-1 and E't represent the position of firm E if it was efficient (on the frontier), implying using the same amount of their inputs, they could have produced as high as their peers, which produced on the frontier (i.e., firms A and B in time *t-1*, and firms B and D in time *t*). The distance function is the ratio of OEt-1/OE't-1 or OE_t_/OE't in time *t-1* and *t*, respectively. Such a ratio varies between **zero** (the most inefficient firm, located on the origin, point O) and **one** (the most efficient firm, located on the frontier of the time).

Similarly, the distances of a Firm from the frontier of the next/previous period can also be defined as shown in Equation (1) above. All four distance measures are shown in Fig. 2, helping to appreciate the implications of the *Malmquist Index*. Thus, the value outside the square brackets in Equation (1) identifies the relative proximity of the firm to the frontier in two consecutive periods (years *t* and *t-*_*1*_). The magnitude of the square brackets of Equation (1), in turn, captures technological change—frontiers' inward or outward shifts over time.

Coelli, et al. [30] demonstrate how DEA and SFA methods can be implemented to estimate the Malmquist TFP index. If the former, it is specifically labelled *DEA-like Malmquist Index*. Nevertheless, it is worth mentioning that these methods often assume a *Constant Return to Scale* (CRS) technology. According to Grifell-Tatjé and Lovell [31], this assumption avoids the interpretation problem encountered with TFP changes when *Variable Return to Scale* (VRS) technology is supposed. In the case of VRS, the estimated TFP changes using this approach may not correctly reflect the TFP gains or losses (see Coelli, et al. [30]. In this method, one should solve four distinct Linear Programming (LP) to estimate the constituents of Equation (1), written in Equations (Equation 2), (Equation 3), (Equation 4), (Equation 5) below. Therefore, for a typical ith power plant, four *d*_*o*_*(.)*s can be measured across two successive periods, *t-1* and *t*:

(Equation 2) $$\begin{array}{l} {\left[d_{o}^{t}\left(y_{t},x_{t}\right)\right]}^{-1}=\max_{\Phi ,\lambda }\Phi \\ \begin{array}{ccc} st & & -\Phi y_{it}+Y_{t}\lambda \geq 0\\ & & x_{it}-X_{t}\lambda \geq 0\\ & & \lambda \geq 0 \end{array} \end{array}$$

(Equation 3) $$\begin{array}{l} {\left[d_{o}^{t-1}\left(y_{t-1},x_{t-1}\right)\right]}^{-1}=\max_{\Phi ,\lambda }\Phi \\ \begin{array}{ccc} st & & -\Phi y_{it-1}+Y_{t-1}\lambda \geq 0\\ & & x_{it-1}-X_{t-1}\lambda \geq 0\\ & & \lambda \geq 0 \end{array} \end{array}$$

(Equation 4) $$\begin{array}{l} {\left[d_{o}^{t}\left(y_{t-1},x_{t-1}\right)\right]}^{-1}=\max_{\Phi ,\lambda }\Phi \\ \begin{array}{ccc} st & & -\Phi y_{it-1}+Y_{t}\lambda \geq 0\\ & & x_{it-1}-X_{t}\lambda \geq 0\\ & & \lambda \geq 0 \end{array} \end{array}$$

(Equation 5) $$\begin{array}{l} {\left[d_{o}^{t-1}\left(y_{t},x_{t}\right)\right]}^{-1}=\max_{\Phi ,\lambda }\Phi \\ \begin{array}{ccc} st & & -\Phi y_{it}+Y_{t-1}\lambda \geq 0\\ & & x_{it}-X_{t-1}\lambda \geq 0\\ & & \lambda \geq 0 \end{array} \end{array}$$

Where symbolisation is characterised by:

*y*_*it*_ is a M × 1 output variables' vector for the ith firm in the tth year

*x*_*it*_ is a K × 1 input variables' vector for the ith firm in the tth year.

*Y*_*t*_ is a N × M matrix of output variables for all N firms in the tth year.

*X*_*t*_ is a N × K matrix of input variables for all N firm in the tth year

λ is a N × 1 vector of weights; and.

Φ is a scaler.

Several software packages can solve these LPs and calculate the distance functions and, ultimately, the *Malmquist TFP index* by decompositions (i.e., efficiency and technological changes). However, we developed our *macro codes* in *Microsoft Excel* to solve the above LPs with Excel's built-in Solver. We used the *Simplex approach* in solving the LPs, which is faster than other algorithms. We also found that using Excel's *macro coding* makes it more practical in managing unbalanced panel datasets like ours. Furthermore, using Excel makes drawing all needed graphs and tables easier. All raw data, models' codes, and Excel results will be shared upon request.

In this paper, to assess whether COVID-19 has significantly affected the estimated efficiency and productivity indicators, we also use the Analysis of Variance (ANOVA) method—a well-established methodology for scientific inferences (Spiegel et al., 2000, pp. 314–324). For instance, Färe, et al. [32] and Atkinson and Halvorsen [33] were among the first studies using ANOVA to statistically assess the impacts of privatisation on the power sector's performance.

## 4 Data

We have collected available input-output data of all power-generating firms in Oman as our DMUs, listed in Oman's Stock Exchange Market. The panel dataset starts from the 1st Quarter of 2015 to the 4th Quarter of 2021 for 12 electricity generation or desalination firms—the longest available period. Selected firms include: (i) Acwa Power; (ii) Al Kamel Power; (iii) Al Suwadi Power; (iv) Albatinah Power; (v) Sembcorp Salalah; (vi) Sharqiyah Desalination; (vii) SMN Power; (viii) Sohar Power; (ix) Phoenix Power; and (x) MCT Desalinate; (xi) MCT Desalinate; and (xii) Musandam Power. These firms collectively produced far above 60 % of electricity generated in Oman (APSR 2021) and have sample power plants from all areas of Oman's Power Sector (i.e., MIS, DPS, and RAEC).

However, the panel is not balanced because some firms have been established recently or recently listed in the stock exchange market. For example, Phoenix Power (the 9th firm) data is available from the 2nd Quarter of 2015 onward. Further, we have data for MCT Desalination since the 4th Quarter of 2017, Dhofar Generating since the 3rd Quarter of 2018, and Musandam Power since the 4th Quarter of 2019. The first eight firms have data for the entire 24 quarterly periods. It is also worth mentioning that the ESI reform was introduced in 2004 and further fine-tuned in 2014. Unfortunately, there are no data available before 2015 in Stock Exchange Market database for electricity firms. Therefore, it is unfortunate that our database does not allow us to compare firms' performances before and after market reform. Nonetheless, we can still assess the performance of the post-reform ESI since 2015.

In addition, we have noted that the quarterly financial data are cumulative. That implies that the 2nd Quarter includes figures from the 1st Quarter, and ultimately the 4th Quarter includes all previous quarters. That means the 4th Quarter is the annual data. However, we decided not to miss any quarterly data as our methodology allows for quarterly and yearly benchmarking of all firms. However, we noticed seasonality in quarterly graphs due to the cumulative data if the periods are quarters. Hence, instead of visualising consecutive quarters as estimation periods, we are comparing each Quarter with the same Quarter of the last year, etc. It means our estimation periods are yearly, with four quarterly observations for each firm. This keeps quarterly observations but presents year-to-year performance comparisons. In this way, we implicitly allow for some extent of data noise. This is while, as we mentioned earlier, the DEA is generally a deterministic approach.

All monetary variables are adjusted with the quarterly CPI to be in real terms. For this purpose, we firstly obtained the monthly CPI from the National Centre for Statistics and Information (NCSI) website and then calculated the quarterly CPI. We have used the same base year in NCSI—the year 2012 (NCSI 2022). We know that it would be ideal to obtain the physical amount of energy inputs (e.g., the volume of natural gas inputs) and the number of employees in each firm. Such data are used in most previous studies (see Section 2). However, in the structure of the Stock Exchange database, such data are not available. Nevertheless, the monetary values of such variables are already embedded in total general and administration expenses or total cost of sales. Thus, we would be safe in comparing like-with-like for all firms.

We have developed four input-output models for our analysis. These models' configurations are presented in Table 2a. The interpretations of TFP are also indicated. Further, definitions of variables as inputs or outputs are listed beneath the Table. Table 2b also presents an overall summary statistic of Input-Output variables used in this study (in Omani Rials).

**Table 2a:** Input-output Configuration of four models.

Model	#Inputs	Inputs	#Outputs	Outputs	Interpretations of higher TFP
1	2	X_1_, X_3_	1	Y_1_	Given the same inputs (costs), more energy sales: higher profit
2	3	X_1_, X_2_, X_3_	1	Y_1_	Given the same inputs (costs), more energy sales: higher profit
3	3	X_1_, X_2_, X_3_	2	Y_2,_ Y_3_	Given the same inputs (costs), higher ROA & ROE: better use of Assets and Equities
4	3	X_1_, X_2_, X_3_	3	Y_1,_ Y_2,_ Y_3_	Given the same inputs (costs), more energy sales & ROA/ROE: higher profit and better use of Assets and Equities

Variables:	Definitions
X_1_:	Total Assets (OMR)
X_2_:	Total general and administration expenses (OMR)
X_3_:	Total Cost of Sales (OMR)
Y_1_:	Sale of Energy (OMR)
Y_2_:	Return of Asset (ROA)
Y_3_:	Return of Equity (ROE)

**Table 2b:** Summary statistic of input-output variables (‘000 OMR).

	Variables	Average	Min	Max	STDEV
Inputs	Total cost of Sale	22644.632	23.930	102285.319	21077.789
	Total general and admin. exp.	598.350	20.729	15739.530	1125.519
	Total Assets	213533.937	7645.429	649234.720	152165.139
Output	Return on Assets (ROA)∗	0.642	0.086	1.384	0.306
	Return on Equity (ROE)∗	0.153	0.001	0.399	0.081
	Sale of energy	32144.192	1377.918	134975.069	29072.004

∗ These variables are financial ratios.

## 5 Empirical results

A selection of the most revealing results is presented in this Section. We first present the overall performance captured by the geometric average of all twelve TFP indices obtained from all four Models. These indices can be interpreted as the overall productivity of the power generation sub-sector of the Omani ESI because they reflect the dominant supply of electricity in the Sultanate. We then deliver individual firms' performances, representing the most inclusive input-output configuration.

**Fig. 3:**
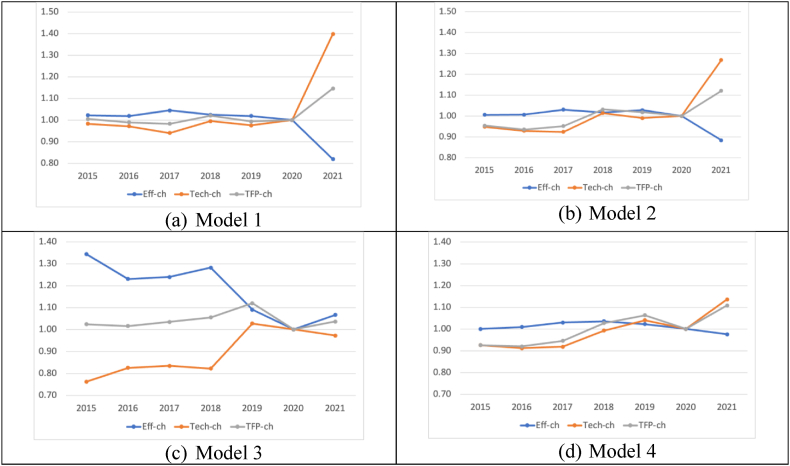
Weighted Average TFP of all electricity firms calculated by Four Models.

Fig. 3 best depicts the dynamics of all twelve firms' overall TFP indices (the grey lines) calculated from the four models (i.e., Models 1–4) by their decompositions. The decompositions refer to the efficiency changes (the blue lines) and technological changes (the orange lines), as described in Section 3. The arbitrarily selected base year for TFP indices in these visualisations is the year 2020. Perhaps, it would have been ideal to choose 2015 as the base year. However, as noted in Section 4, not all firms have TFP estimates between 2015 and 2018. Thus, out of the three remaining years, 2020 is arbitrarily chosen for this purpose.

Fig. 3 also demonstrates the sensitivity of aggregate TFP indices against the Model's input/output configurations. One should also be mindful of the interpretation of TFPs and efficiency scores corresponding to each model configuration (see Table 2a above). For instance, Model 2 has only one additional input variable (total general and administration expenses) as compared to Model 1, which has two inputs (i.e., Total Assets and Total Cost of Sales) and one output (Sale of Energy). There is little difference between the pattern of TFP changes in panels (a) and (b) of Fig. 3. Such a pattern is irrespective of the magnitude of changes.

In terms of efficiency scores, on average, based on Models 1 and 2, all twelve firms were collectively highly efficient between 2015 and 2020. All firms collectively and, on average, got the closest distance from the estimated frontiers in 2017. Still, the differences between the average efficiency scores were negligible during 2015–2020. Both Models interestingly imply a minor drop in the average efficiency scores of all twelve firms in the aftermath of the COVID-19 Pandemic moving from 2019 to 2020. A much more significant efficiency falls occurred in 2021. More specifically, Model 1 and Model 2 reveal that the entire power generating system was some 12–18 per cent less efficient in 2021 than in 2020 (see the blue lines in Fig. 3, panels (a) and (b)).

Regarding technological changes (i.e., shifts in frontiers), Model 1 and Model 2 reveal minor inward shifts in the best practice frontiers in 2016 and 2017, followed by an outward shift in 2018. There has been a significant outward shift in 2021 after the COVID-19 Pandemic (see the orange lines in Fig. 3, panels (a) and (b)). We will discuss the likely reasons behind such a pattern of changes shortly (after the results of all four Models are completed) based on additional information we have collected from the industry.

Models 1 and 2 imply that the TFP indices have been steady and close to one over 2015–2020 while considerably improving in 2021 (12–15 % better than the base year). Such a stable performance is because efficiency and technological changes have moved in opposite directions and offset one another's effects. While outward shifts of the best practice frontier represent better technological achievements, on average, firms collectively got farther from the best practice frontier in 2021. Thus, our analysis suggests that power-generating companies in Oman have performed much better in 2021 than in 2020. Even though the Pandemic was not over in 2021, the country adapted to the situation and moved forward.

The difference between Model 2 and Model 3 is in their output variables. The latter has ROA and ROE financial ratios as the output variables. That implies that a firm performs better if its Assets/Equities are utilised more efficiently (the higher ROA and ROE). Thus, panels (b) and (c) of Fig. 3 are significantly different because RAO and ROE variables have no units—unlike the Sale of Energy in the Omani currency (i.e., OMR) with the exact nature of the input variables (Costs in OMR). Finally, Model 4 has only one more output (i.e., Sale of Energy) than Model 3 with the same input variables. The interpretation of TFP and efficiency in Model 4 imply that a firm performs better when, given the same inputs (costs), it obtains more energy sales and higher ROA/ROE. In this sense, the pattern of TFP changes (and its components) in panel (d) of Fig. 3 is more like panels (a) and (b). This observation implies the importance of the Sale of Energy as an output variable.

Panel (c) of Fig. 3 suggests that, based on Model 3, there has been a significant technological shift (the orange line) in the generation subsector's best practice frontier from 2018 to 2019. Interestingly, based on Model 3, there was an inward shift in the best practice frontier after COVID-19 in 2021. This fact is unlike Model 1 and Model 2 (even Model 4, yet to be discussed). Nonetheless, Model 3 suggests that the relative efficiency (the blue line) has changed in the opposite direction as the technological shifts (the orange line), making the overall TFP change pattern very similar to those of Model 1 and Model 2—improving regardless of the magnitudes. In the post-COVID-19 period (i.e., the year 2021), Model 3 suggests that the power generation sub-sector has shown 7 % higher efficiency, while there has been some 3 % inward shift in the best practice frontier in 2021 as compared to 2020. This result reflects that the industry is getting closer to the best practice. This implies an overall 4 % improvement in TFP compared to 2020.

Model 4 reveals that the power generation sub-sector has shown 3 % lower efficiency (relatively getting farther from the best practice frontier), while there has been some 14 % outward shift in the best practice frontier in 2021 compared to 2020. This implies an overall 11 % improvement in TFP in 2021 compared to 2020. From 2015 to 2020, the efficiency changed marginally, and the best efficiency was experienced in 2018, some 4 % higher than in 2020. Regarding technological changes (shifts in the best practice frontier), there were minor inward shifts in 2016, followed by three outward shifts in 2017, 2018 (most significant change) and 2019. Then, an inward shift occurred in 2020, which is the likely outcome of COVID-19. This outcome was not observed in Model 1 or Model 2 but seen in Model 3, implying that the Pandemic's recession left more Assets/Equities (captured in the output variables used in Models 2 and 3) idle, clearly due to the Pandemic. This is followed by a significant outward shift in 2021 (the highest in the entire 2015–2021 period), which was also observed in Model 1 and Model 2 (but interestingly not in Model 3).

More specifically, the cumulative TFP index for all 12 firms based on Model 4 was 0.925 in 2015. This measure reached its highest level in 2019, equal to 1.063. This shows approximately 15 % growth that smoothly and steadily took place, with an average annual growth rate of 3.5 % per annum over four years. The TFP index then drops to 1.0 in 2020—a negative 6 % growth. However, it raised to 1.108 in 2021—an 11 % growth.

From the results of this study—summarised in Fig. 3—the predominant observation is that COVID-19 negatively affected the sector's TFP in 2020 but quickly regained its strength in 2021. The breakdown of the aggregated TFP index of the sample firms verifies that the decline in TFP in 2020 (observed in Models 1–4) is triggered by both a fall in average efficiency scores and an inward shift in the estimated best practice frontier (except Models 1 and 2, which are missing more variables). These are probably the consequences of the recessionary effects of the Pandemic. While average efficiency scores continue to fall (except Model 3), an outward shift in the frontier helps a rapid revival in 2021. Such technological improvements may be due to firms' (at least some) investments in more efficient technologies (e.g., technical upgrades). For instance, it is observed that the fuel utilisation of the gas operating units of Nama Holding has improved by 2.82 % due to the upgraded plants (e.g., Sohar 3 and Ibri), which started operation in May 2019 (Nama 2020). This may also be due to some firms' better operational use of online technologies (reduced operating costs for working remotely). In most cases, significant outward shifts (the orange lines) have started from 2018/2019 onward, far prior to the Pandemic.

We contend that, out of these four Models, Model 4 provides the most realistic performance of the power-generating subsector because it is better specified—not excluding any variables. Whereas the input variables are the same as Model 2 and Model 3, the output variables comprise ROA, ROE, and Sale of Energy. The interpretation of TFP is also more indicative (See Table 2). This Model combines the contrasting outcomes between Model 2 and Model 3. Thus, we will use this Model's results only for the firm-specific results. Details of the firm-specific results from other models can be shared upon request.

At individual firm levels, we present the results statically (year-by-year and quarter-by-quarter) and dynamically (over the entire period). The static relative efficiency in each estimation period is essential in determining the best practice frontier in each year (corresponding to LPs of Equation (2) or 3). In the frontier approach, the relative efficiency in each year/period varies between 0 and 1. Firms located on the best practice frontier take an efficiency score of 1. Table 3 shows all twelve firms' relative static efficiency scores each year and Quarter. The colour code varies from dark green (efficiency score of one) to white (middle efficiency scores, 0.5), then to dark red (zero efficiencies). Table 3, for instance, shows that Al Kamal and Musandam Power have always been on the frontier. As mentioned earlier, Musandam Power joined the generation subsector in the 4th Quarter of 2019. Apart from these two firms, Acwa Power, Albatinah Power, Sembcorp Salalah, Sharqiyah Desalination, SMN Power, and Sohar Power have occasionally been on the estimated best practice frontiers on a quarter-by-quarter basis. On an annual basis (the 4th Quarters), though, Al Kamal, Sembcorp Salalah, SMN Power, and Musandam Power have always been on the frontier, peering at other firms—being used for calculating their annual efficiency scores. The firms that have consistently performed below the best practice include Al Suwadi Power, Phoenix Power, MCT Desalinate, and Dhofar Generating.

**Table 3:**
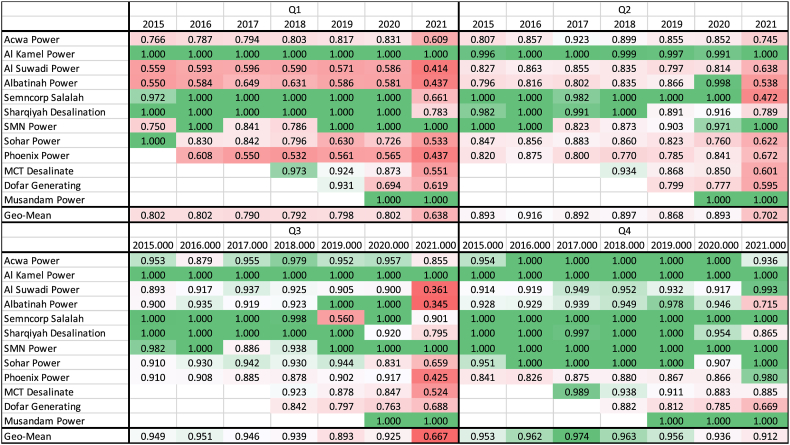
Static relative efficiency in each year and by quarters.

The 4th Quarter covers the completed financial data for each year. Hence, we have used the fourth Quarter's performance as the annual performance of power generating companies. The greener colour (and the lesser red) of this quadrant of Table 3 can be considered a good indicator for the electricity generation subsector in Oman, implying the average annual efficiency score of the entire power-generating subsector is close to the best practice (efficiency of one). This observation was mentioned earlier when showing the blue line in Fig. 3 panel (d) is consistently close to one between 2015 and 2021. However, we mentioned earlier in Section 3 that the *Malmquist TFP index* assumes a constant return to scale (CRS) technology to avoid any interpretation problem of dynamic changes in TFP (Section 3). This may not be entirely useful because the Model may ignore the economies of scale that may apply to larger firms' efficiencies.

Nevertheless, the poorest annual performance is experienced by Dhofar Generating company (0.67), followed by Albathina Power (0.72) in the 4th Quarter of 2021. We can also observe that more firms underperform in the First Quarter of each year as we can see more red (lesser green) colours in the first quadrant of Table 3. The poorest quarterly performance has been observed in Albatinah Power (0.34), followed by Al Suwadi Power (0.36) in the 3rd Quarter of 2021. Interestingly, the same firms' static efficiency scores were in a much better position in the 4th Quarter of the same year (0.72 and 0.99, respectively).

**Fig. 4:**
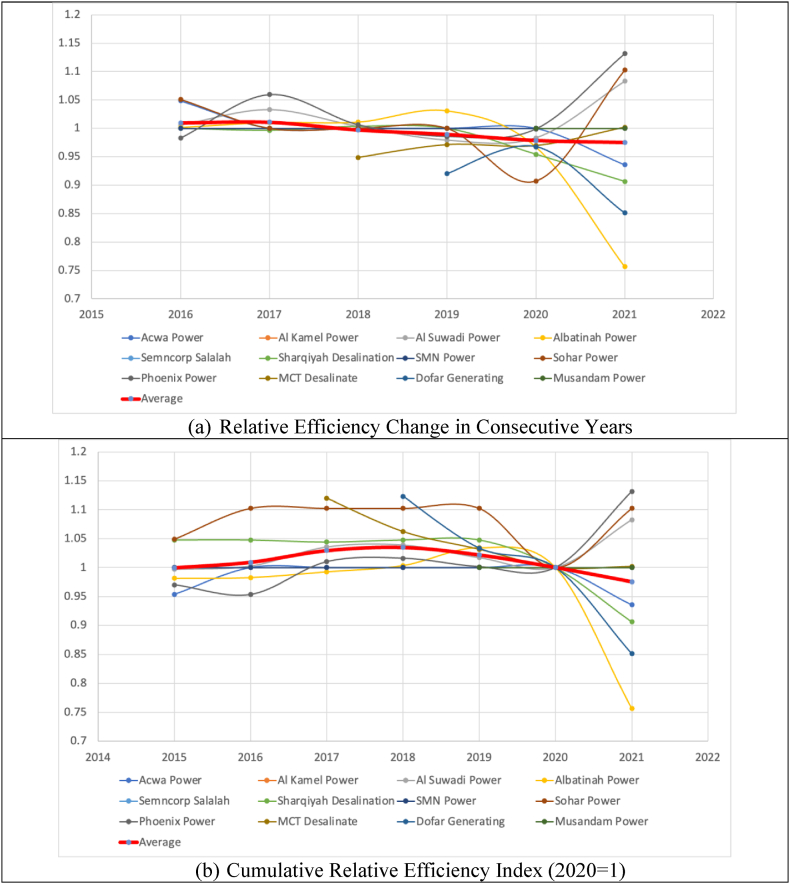
Dynamics of efficiency changes by firms, calculated by Model 4.

Fig. 4 panel (a) demonstrates the dynamics of changes in the relative efficiency scores, i.e., the ratio outside the square brackets in Equation (1). Fig. 4 Panel (b), in turn, shows the cumulative index made from Panel (a), while 2020 is considered the base year. In this way, Fig. 4 Panel (a). offers each firm's relative proximity to the industry's frontier in each period compared to its previous period, whereas Fig. 4 Panel (b) shows a similar notion, comparing each firm's relative proximity to the industry's frontier in a period with the base year (2020 = 1). For example, Al Kamal Power and AL Musandam, which have always been on the year-to-year best practice frontiers (see Table 3), also remain at score 1 in Fig. 4 because they have zero distance from the best practice frontier in all periods. The thick red line shows the geometric average of all twelve firms (the industry's average estimate). The average line in Fig. 4 Part (b) in red was previously shown in Fig. 3 Part (d) in blue.

**Fig. 5:**
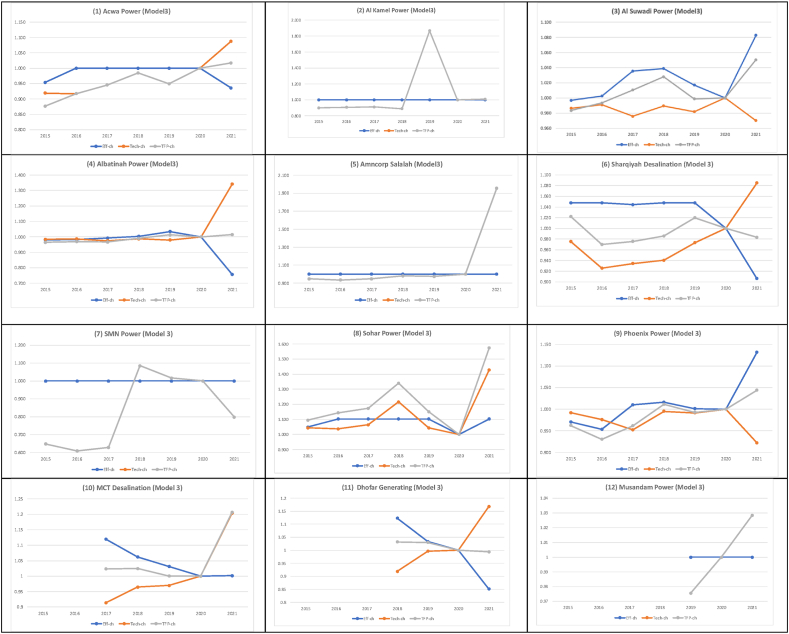
Cumulative TFP indices by their decomposition for the selected firms (model 4).

At firms' levels, Fig. 5 shows the dynamics of cumulative annual TFP and efficiency changes for all twelve electricity firms based on Model 4. Like Fig. 3, the blue line offers the relative proximity of firms to the estimated frontiers over time (2020 = 1), while the orange line reflects the technological changes. As noted earlier, Al Kamal, Smncorp Salalah and SMN Power have always been on frontier curves for annual data points (the 4th Quarter). Musandam Power has also demonstrated a similar trend from 2019 onwards. Thus, the blue lines for these firms remain constant on score one efficiency, showing no change in their efficiency scores. As for other firms, the blue line may be higher or lower than one, indicating their relative positions against the base year.

Only focusing on 2021 compared to 2020, Phoenix Power, Sohar Power, Al Suwaidi Power, and MCT Desalination (in descending order in terms of the magnitudes of changes) have shown relative improvement in their efficiency scores despite the Pandemic. The latter is very negligible. Quite the contrary, Albatinah Power, Acwa Power, Sharqiyah Desalination, and Dhofar Generating (again in descending order in terms of the magnitudes of changes) have shown a significant drop in their efficiency from 2020 to 2021. These observations are even more noticeable in Fig. 4, panel (b). None of these changes can be caused by COVID-19 indeed. We contend such individual efficiency fluctuations would be about firm-specific behavioural matters (e.g., management). Similar observations are also valid for the efficiency changes prior to the Pandemic (i.e., 2015–2019). Considering the changes from 2019 to 2020, we note that except for Acwa Power and Phoenix Power (and, of course, firms with consistently being on the frontier), the remaining six firms have experienced a substantial decrease in their efficiency scores moving from 2019 to 2020 (see also Fig. 4, panel (b)). Thus, such efficiency declines can undoubtedly be associated with COVID-19.

In Fig. 5, the firm-specific observations regarding the technological changes (the orange lines) over the entire estimation period (2015–2021) are diverse. Only five firms (out of 12) predominantly and consistently show that they are positively affected by technological improvements (upward shifts). These firms include Acwa Power, Sharqiyah Power, MCT Desalination, Dhofar Power, and Musandam. Other firms are affected differently. We observe a mixture of positive and negative impacts on firms' TFP performance caused by inward/outward shifts in best practice frontiers. These imply the occurrence of recessionary or expansionary shocks at Omani ESI. When focusing on 2021, we observe eight firms are positively affected and three firms are negatively affected by the technological shift that took place in 2021. One firm remains unaffected. These imply that technological advancements (upward frontier shifts) have positively impacted many firms in 2021. This fact was also observed in Fig. 3 Panel (c).

Finally, as we mentioned in Section 3, we apply ANOVA tests to statistically identify whether COVID-19 has significantly affected the performance of the sample firms. The typical *null hypothesis* (*H*_*0*_) states that the mean of estimated indicators in pre- and post-COVID periods are equal. Given the observations, whenever the F statistic is larger than the critical F values (at a certain level of significance), one can reject *H*_*0*_, implying that COVID-19 has statistically affected the performance indicators. Table 4 summarises ANOVA tests against measured indicators averaged for the entire firms.^2^

**Table 4:**
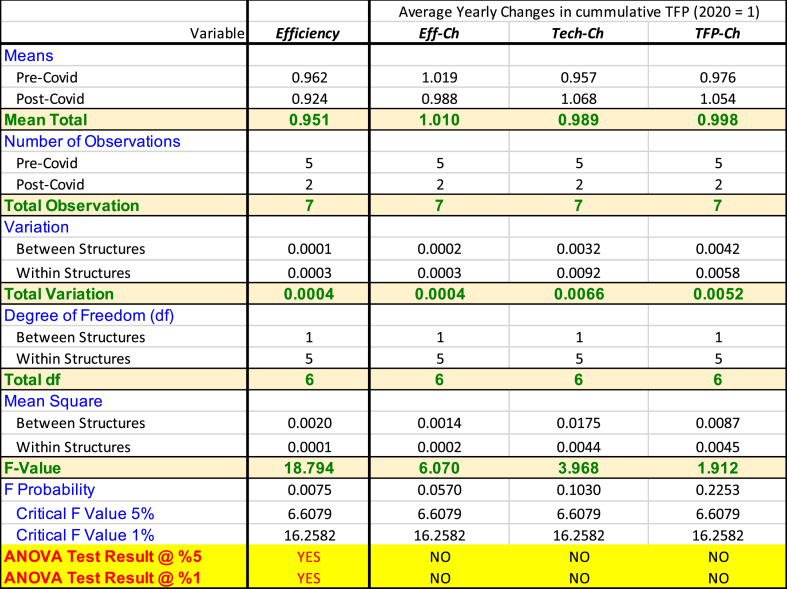
Summary of ANOVA Hypotheses Tests.

The ANOVA test results predominantly reveal that COVID-19 has no impact on cumulative TFP indices. However, in terms of static efficiency scores, the test suggests that the mean efficiency scores in the post-COVID period are significantly lower than in the pre-COVID period. These results are consistent with the earlier analysis provided in this Section above. For instance, we noticed that the average static efficiency score is lower during the post-COVID period (see Table 3, the colour tends toward darker red in 2020 and 2021). We can hence firmly say that the outward shifts in the best practice frontiers have been the leading cause behind the rapid recovery in TFP indices in 2021.

## 6 Conclusions & policy implications

This study aimed to analyse the changes incurred in productivity and efficiency measures within the generation sector of the Omani electricity industry. We presented the preliminary results of this study at the 44th IAEE International Conference [4], but this paper presents extensive results. An overarching finding is that the aggregated TFP index exhibited a consistent upward trajectory from 2015 to 2019. Subsequently, TFP measures declined in 2020, followed by a swift rebound in 2021, regardless of their magnitudes. This observation reinforces the inference that COVID-19 adversely impacted the industry's overall performance in 2020 but experienced a speedy recovery in 2021. The overall Total Factor Productivity (TFP) index decomposition analysis for all businesses indicates that the decline in TFP is attributed to a decrease in average efficiency scores and a shift towards a lower predicted best practice frontier in 2020. These are probable consequences of the recessionary effects caused by the Pandemic. However, despite the decline in average efficiency ratings, a change in the frontier is aiding a rapid comeback in 2021. We argue that the expansion of boundaries can be attributed to practical and suitable technology advancements, particularly in the units owned by the enterprises in the sample, beginning in 2018.

We have noted that, out of the four models, Model 4 provides the most realistic performance of the power-generating subsector because it is better specified—not excluding any variables. The interpretation of TFP is also more indicative because it combines the contrasting outcomes between Model 2 and Model 3. It implies that, given the same inputs (costs), a higher Sale (more profit) and better use of Assets/Equities would refer to a more productive firm. As a result, we present our conclusions based on Model 4, both at aggregate and firm-specific levels. Key findings are summarised below (further results at firms' levels are presented in Appendix A1).

• The ANOVA test reveals that COVID-19 has no impact on cumulative TFP indices but negatively affects the industry's overall efficiency scores.
• In 2021, the average TFP of the power-generating subsector had an overall 11 % improvement compared to 2020.
• From 2015 to 2020, the relative efficiency measures have increased marginally, reaching their highest level in 2018, some 4 % higher than the base year (2020).
• Regarding technological changes (shifts in the best practice frontier), there were minor inward shifts in 2016, followed by three outward shifts in 2017, 2018 (highest) and 2019.
• An inward shift occurred in 2020, which is the likely outcome of COVID-19.
• Interestingly, this result was not observed in Model 1 or Model 2 but in Model 3 as well.
• Such differences in the Model's results imply that the industry has performed less productively due to the Pandemic because some firms have gotten farther from the best practice frontiers.
• Such firms were less efficient in using their Assets/Equities—the output variables used in Models 3 and 4 only.
• More specifically, based on Model 4:
  o The cumulative TFP index for all 12 firms was **0.925** in 2015, smoothly and gradually growing by 15 % during 2015–2019, reaching its highest level in 2019, i.e., **1.063.**
  o The TFP index dropped to **1.0** in 2020—a 6 % fall—due to COVID-19.
  o The TFP index, however, recovers to **1.108** in 2021—an 11 % growth.

What are the policy implications of this analysis? Here, based on our best judgments and some other information we have obtained, we briefly offer critical policy implications of this study alongside ways to improve future studies in this line of research.

Apart from the observed inward (recessionary) and outward (boom) shifts related to economic business cycles, we pose the question if there have been any genuine structural improvements (i.e., technological advancement) in the Omani ESI. Given the current analysis, we can certainly answer “yes” to this question. Among four firms consistently being on the frontier, two have significantly and consistently shown higher TFPs over time. These firms comprise *Sembcorp Salalah* and *Musandam Power* (see Fig. 5). These observations imply that at least these two firms have been able to reach what were unattainable data points, given previous years' technologies. *Sohar Power* also performed superbly in 2021 (also during 2017–2018) as their technological and efficiency changes positively reinforced one another. These three firms have hence shown the most remarkable performance most of the time. They have been among the firms pushing the boundaries for a better future for the industry. However, the question is what they have done to demonstrate such an outstanding performance needs to be further analysed by quantitative, qualitative, and institutional measures. As identified role models, other poorly performed peers can take similar measures that these firms have implemented to improve their productivity.

Many details within the twelve power-generating firms should be further analysed and scrutinised to identify the rationales behind the efficiency and technological changes. For example, the Omani ESI is dominantly fuelled by natural gas. We have noticed that specifically combined cycle gas turbine (CCGT) units have been added to generating capacity of some of the firms during the period of this study. Any upgrades from the gas turbine (GT) to CCGT or installing new modern CCGTs or other advanced technologies can cause a shift in the frontiers. GT's fuel efficiency is around 20–40 %, steam turbines (STs) are about 30–35 %, while advanced CCGT units' fuel efficiency can be as high as 60–65 %. The so-called hybrid power plants' fuel efficiency may even exceed 70 % (Boyce 2012). Accordingly, advanced CCGTs may significantly reduce the operating costs of power generation captured in our models.

Specifically, we have noticed that Sembcorp Salalah recently commissioned its CCGT unit (445 MW). After detailed engineering planning, Sembcorp Salalah chose to integrate five GTs with five units of heat steam generators and two STs in a CCGT configuration to achieve optimal energy production efficiency (Sembcorp 2022). Further, Sohar Power has also commissioned a CCGT power plant with a 585 MW installed capacity and a 150,000 m^3^/d desalination plant located in the North Al Batinah Governorate of the Sultanate. The site is chosen to be located near the primary natural gas transmission system, the electricity transmission, the water grid network, and other process industries (Sohar Power 2021). This decision may have caused substantial savings in the operational costs of Sohar Power. Such information endorses our findings and reveals one of the main rationales behind these firms' remarkable performance—experiencing significant technological shifts.

Having said the above, we are confident that policymakers of Omani ESI—with more in-depth knowledge of the industry—might be better guided by this study's results to set specific technological standards to improve the whole system's efficiency or shift the frontier into a better position. Thus, we believe this research benefits industry experts, practitioners, and policymakers of Omani ESI, adding policy insights to their industry knowledge.

It is worth mentioning that, while being very useful, one should always be aware of the limitations of frontier analysis. This approach cannot precisely pinpoint what the sources and causes of changes are. Hence it does not indicate per se what measures must be implemented by poorly performed firms to fix their problems. For these purposes, complementary *normative analyses* (e.g., additional assessment frameworks, experts' views/opinions, or institutional analysis) should be coupled with frontier analysis to draw practical policy implications. For instance, we implemented ANOVA tests and pointed out that COVID-19's impact on the industry's TFP has been mainly insignificant because there have been outward shifts in the best practice frontier. We revealed that some firms' genuine investments in modern technologies are due to sound investments making the industry more cost-effective and fuel-efficient, at least in some leading firms.

Therefore, regarding the specific policy implications of this research, the jury is still out. This study is dominantly a *positive analysis* revealing factual changes in productivity and efficiency measures. It holds enough contributions as empirical research. However, more contributions in this area of research would be possible by the following suggestions. Using more inclusive input-output model configurations may shed more policy insight on this matter. Using appropriate assessment frameworks such as hypothesis tests against specific qualitative measures (structural, regulatory, or technological changes) can bring more helpful policy insights. For instance, Aghdam (2011) have developed an objective institutional assessment framework using hypothesis tests to determine whether the Australian electricity market reform has contributed to the industry's productivity/efficiency gains. A similar analysis can be implemented in the context of Omani ESI. Furthermore, benchmarking the Omani firms against a broader GCC region or beyond would be more revealing and practical. These are some avenues for follow-up research studies for the Omani ESI.

## CRediT authorship contribution statement

**Reza FathollahZadeh Aghdam:** Writing – review & editing, Writing – original draft, Visualization, Validation, Supervision, Software, Resources, Project administration, Methodology, Investigation, Funding acquisition, Formal analysis, Data curation, Conceptualization. **Sami Al-Kharusi:** Writing – original draft, Validation, Resources, Project administration, Methodology, Investigation, Formal analysis, Data curation, Conceptualization. **Nisar Ahmad:** Writing – review & editing, Writing – original draft, Visualization, Validation, Supervision, Resources, Project administration, Methodology, Investigation, Funding acquisition, Formal analysis, Conceptualization. **Adham Al-Said:** Writing – review & editing, Writing – original draft, Visualization, Validation, Supervision, Resources, Project administration, Methodology, Investigation, Formal analysis, Data curation, Conceptualization. **Bahareh Berenjforoush Azar:** Visualization, Validation, Supervision, Software, Resources, Project administration, Methodology, Investigation, Formal analysis, Data curation, Conceptualization.

## Declaration of competing interest

The authors declare that they have no known competing financial interests or personal relationships that could have appeared to influence the work reported in this paper.

## Appendix A1

At the firm-specific level, the 4th Quarter covers the completed financial data for each year. Hence, we have used the 4th Quarter's performance as the annual performance of power generating companies. The most intriguing firm-specific conclusions include.

• **Al Kamal** and **Musandam Power** have always been on the year-to-year frontiers.
• Apart from the above-two firms, considering quarterly data points, **Albatinah Power**, **Sembcorp Salalah**, **Sharqiyah Desalination**, and **SMN Power** have occasionally been on the estimated best practice frontiers.
• On annual estimation (the 4th Quarter), **Sembcorp Salalah** and **SMN Power** have also been on year-to-year best practice benchmarks.
• The poorest annual performance is experienced by **Dhofar Generating company** (0.67), followed by **Albathina Power** (0.72) in the 4th Quarter of 2021.
• More firms underperform in the 1st, 2nd, and 3rd Quarters each year than in the 4th Quarter.
• The poorest quarterly performance has been observed in **Albatinah Power** (0.34), followed by **Al Suwadi Power** (0.36) in the 3rd Quarter of 2021.
• Only focusing on 2021 compared to 2020:
  o **Al Suwaidi Power**, **Sohar Power**, and **Phoenix Power** have improved their **relative** efficiency scores despite the Pandemic.
  o **Acwa Power**, **Albatinah Power**, **Sharqiyah Desalination** and **Dhofar Generating** have performed relatively poorer efficiency in 2021 in the aftermath of the Pandemic.
• Regarding individual firms' TFP indices, **Sembcorp Salalah** and **Sohar Power** show the most remarkable improvements in their 2021 TFPs: technological advancements further boosted by their relative efficiency improvements (getting closer to the best practice frontier).
• For most other firms, these two effects have offset each other, causing a mixed impact on their TFP indices.

## Footnotes

1 TITLE ((electric* OR “power generation” OR desalin*) AND (efficiency OR productivity)) AND TITLE-ABS-KEY ((“data envelopment” OR “stochastic frontier” OR “technical efficiency” OR “allocative efficiency” OR “scale efficiency” OR “total factor productivity” OR malmquist OR parametric OR “non parametric”)) The search has been carried out in May 2022.

2 Similar ANOVA tests are performed at individual firm levels and can be shared upon request.
